# 163. Invasive Cryptococcal Infection in Hospitalized HIV and Non HIV Patients: A Nationwide Analysis of Risk Factors and Outcomes

**DOI:** 10.1093/ofid/ofaf695.058

**Published:** 2026-01-11

**Authors:** Armin Safdarpour, Chukwuemeka Umeh, Chawki Harfouch, Pranav Barve, Emily Kim, Vikas Patel

**Affiliations:** KPC Hemet Global Medical Center, Menifee, CA; KPC Hemet Global Medical Center, Menifee, CA; Loma Linda University Medical Center, Murrieta, California; KPC Hemet Global Medical Center, Menifee, CA; KPC Hemet Global Medical Center, Menifee, CA; KPC Hemet Global Medical Center, Menifee, CA

## Abstract

**Background:**

Invasive cryptococcal infection ICI is a fungal disease with an estimated global mortality of up to 74 %, occurring predominantly in persons living with HIV ( PLWH). However, it is increasingly seen in individuals with other immunocompromising conditions or no obvious risk factors ( non-PLWH). Interestingly, non-PLWH with invasive cryptococcal infections are diagnosed later and experience higher morbidity and mortality. We aim to compare the outcomes and predictors of mortality in PLWH and non-PLWH cryptococcal infections.

**Methods:**

We retrospectively analyzed data of patients 18 years and older hospitalized with a primary or secondary diagnosis of ICI admitted in United States hospitals from January 2017 to December 2021 using the National Inpatient Sample (NIS) data.

**Results:**

We identified 13,615 patients with a discharge diagnosis of ICI, of which 10,130 (74.4%) were males. 6,950 patients (51%) were PLWH, and 1,430 (10.5%) died during the hospital admission. The all-cause mortality was 7.6% in PLWH and 13.6% in non-PLWH. Of the 6,665 ICI cases in non-PLWH, 350 (5.3%) had a documented autoimmune disorders, 930 (14%) were organ transplant recipients, 125 (1.9%) had solid malignant tumors, 335 (5.0%) had leukemia, 710 had diabetes (10.7%), 405 had cirrhosis (6.1%) and 660 (9.9%) had end stage renal disease. 3690 (55.4%) did not have any obvious risk factors.

In the multivariate analysis, non-PLWH were significantly more likely to be females, older, and caucasian (all with P< 0.001) when compared to PLWH. In addition, non-PHLW were more likely to have leukemia, cirrhosis, autoimmune disorder, and be transplant patients (all with P< 0.001). Length of hospital stay as well as inpatient mortality was significantly higher in non-PLWH (P< 0.001), and mortality for both groups was associated with shock upon presentation (P< 0.001). In the analysis of mortality predictors, septic shock was the greatest predictor of mortality in both groups, with a 17 times increased risk of mortality (p< 0.001).

**Conclusion:**

Non-PLWH with ICI experienced higher mortality and length of hospital stay than PLWH, but both groups were at high risk if they presented with shock. Cirrhosis and ESRD negatively impacted non-PLWH and PLWH, respectively.

**Disclosures:**

All Authors: No reported disclosures

